# Effects of Two Antiretroviral Drugs on the Crustacean *Daphnia magna* in River Water

**DOI:** 10.3390/toxics10080423

**Published:** 2022-07-28

**Authors:** Ntombikayise Mahaye, Ndeke Musee

**Affiliations:** Emerging Contaminants Ecological and Risk Assessment (ECERA) Research Group, Department of Chemical Engineering, University of Pretoria, Pretoria 0028, South Africa; mahaye.ntombi@gmail.com

**Keywords:** HIV antiretroviral drugs, Tenofovir, Efavirenz, antioxidant enzymes, biochemical markers, *Daphnia magna*

## Abstract

Antiretroviral (ARVs) drugs are used to manage the human immunodeficiency virus (HIV) disease and are increasingly being detected in the aquatic environment. However, little is known about their effects on non-target aquatic organisms. Here, *Daphnia magna* neonates were exposed to Efavirenz (EFV) and Tenofovir (TFV) ARVs at 62.5–1000 µg/L for 48 h in river water. The endpoints assessed were mortality, immobilization, and biochemical biomarkers (catalase (CAT), glutathione *S*-transferase (GST), and malondialdehyde (MDA)). No mortality was observed over 48 h. Concentration- and time-dependent immobilization was observed for both ARVs only at 250–1000 µg/L after 48 h, with significant immobilization observed for EFV compared to TFV. Results for biochemical responses demonstrated that both ARVs induced significant changes in CAT and GST activities, and MDA levels, with effects higher for EFV compared to TFV. Biochemical responses were indicative of oxidative stress alterations. Hence, both ARVs could potentially be toxic to *D. magna*.

## 1. Introduction

The increase in the use of drugs and advances in analytical capabilities have led to detectable concentrations of pharmaceuticals in variant natural (e.g., surface water, and groundwater) and technical (e.g., wastewater treatment plants (WWTPs), tap water) systems [1,2,3,4]. However, ecological risks for most classes of pharmaceuticals remain largely unknown [4,5]. Among these pharmaceuticals are antiretroviral drugs (ARVs) used in the fight against human immunodeficiency virus (HIV) [6]. To date, HIV remains a major global public health issue with some 37.7 million people living with HIV (PLHIV) worldwide by 2020 [7]. In addition, the prescription of lifelong antiretroviral therapy (ART) to combat high morbidity and mortality has increased dramatically post-2017 [8], with over 30 ARVs from six different classes [9] (Table S1). This indicates the likelihood for an increase of release into the environment. For instance, some 28.2 million PLHIV were on ART as of 30 June 2021 [7] with about 20 tons of ARVs being consumed daily globally, with South Africa accounting for 20% of the total mass (*ca* 4 tons) [2].

At least 90% of PLHIV globally were expected to be on ART by 2020 [8]. Therefore, to attain this target, ARVs should be accessible to all PLHIV. This, in turn, will dramatically increase ARVs consumption, with eventual further increases in their release into the aquatic environments. For example, since the mid-2000s, detected concentrations of ARVs in the ecosystems have dramatically increased in different regions (Table S2). Yet, ecotoxicity studies of ARVs have only been published post 2010 [5,10,11,12,13,14,15,16]. However, these are highly limited given the large number of ARV drugs on the market. Because of increased detection in many countries, at concentrations from the ng/L to µg/L range, and the limited knowledge on the potential to cause adverse effects to the ecosystem, ARVs are now considered to be emerging contaminants (ECs) belonging to the class of Pharmaceutical and Personal Care Products (PPCPs) [17].

Among ARVs, Efavirenz (EFV) and Tenofovir (TFV) are the most prescribed drugs for the management of HIV/AIDS [6]. Post absorption, distribution, metabolism, and excretion (ADME) processes in PLHIV, original ARVs, and their metabolites are excreted from the body through urine or feces [18,19,20], and eventually into the WWTPs [21,22,23], and/or directly into surface waters in areas lacking sanitation infrastructure [24]. For example, 71% of lamivudine (3TC) is excreted via urine, while 80 and 10% of nevirapine (NVP); are excreted in urine and feces, respectively [25,26].

Growing evidence demonstrated that ARVs could potentially pose variant deleterious effects on non-target aquatic organisms at different trophic levels. These include bactericidal [12,27] and mutagenicity effects [28] in bacteria, growth inhibition and/or promotion in algae [28,29], immobilization of aquatic invertebrates [12], bioaccumulation and alteration of antioxidant systems in amphibians [14] as well as histological changes and growth rate inhibition in fish [5,13,15]. Notably, most of these studies were carried out using synthetic media; hence, they are not representative of the actual aquatic environment. To our knowledge, only two studies [13,30] assessed the effects of NVP in fish using borehole water as the exposure medium. This raises an urgent need for more studies in actual environmental matrixes (e.g., river water) to elucidate the mechanisms of ARVs’ toxicity.

Several biomarkers including antioxidant defense mechanism and lipid peroxidation (LPO), have been successfully employed to elucidate the oxidative stress of pollutants in environmental studies [31,32,33,34,35]. Assessment of early responses of enzymatic biomarkers from different metabolic pathways can offer valuable insights into the toxicological effects of pharmaceutical compounds on non-target aquatic organisms [36,37]. For instance, reactive oxygen species (ROS) produced in excess can react with macromolecules and induce protein denaturation, lipid peroxidation, and changes in antioxidant enzyme activities [38,39]. Previously, variant ARVs have been reported to induce ROS production in rats [40] and amphibians [14]. ROS-caused lipid peroxidation was shown to induce biological membranes damage with deleterious consequences at the cellular and organism levels [33,41]. To minimize oxidative damage to cellular components, organisms generally develop antioxidant defense mechanisms [32]. Failure of antioxidant defense mechanisms to detoxify excess ROS production can lead to significant oxidative damage including enzyme inactivation, protein degradation, DNA damage, and lipid peroxidation [42].

Antioxidant enzymes such as catalase (CAT) and glutathione *S*-transferase (GST) detoxify ROS and are considered important antioxidant response systems in invertebrate species [32,43]. Catalase reduces hydrogen peroxide (H_2_O_2_) to water and oxygen [44,45]. Moreover, GST catalyzes the conjugation of glutathione (GSH) with various electrophilic xenobiotics [46], thereby protecting the cells against mutagenic, carcinogenic, and apical effects of the compounds [47].

Despite a dramatic increase in the use of ARVs—especially in Sub-Saharan Africa (SSA), a region with the highest population of PLHIV [48]—their toxicity to aquatic organisms remains poorly quantified and hence forms the aim of this study. The study investigated the toxicity of two first-line ARVs, EFV and TFV, which represent non-nucleoside reverse transcriptase inhibitors (NNRTI) and nucleoside reverse transcriptase inhibitors (NRTI) classes, respectively, on freshwater crustacean *Daphnia magna*, a common freshwater zooplankton highly sensitive to xenobiotics. *D. magna* was chosen as it is frequently used as a test organism in toxicity studies [49] and is also an important species in freshwater food chains as primary consumers.

Here, the specific study objectives were two-fold: to assess (i) apical endpoints (immobilization, and mortality), and (ii) biochemical biomarkers (e.g., CAT, GST, and LPO) responses of *D. magna* exposed to 62.5–1000 µg/L EFV and TFV ARVs for 48 h. The latter objective endeavored to understand the toxicity mechanisms of each ARV type, and linkages to the observed effects at the apical endpoint, and sub-lethal levels. Here, river water was used as the exposure medium as opposed to widely used standardized media (e.g., ASTM or ISO medium) to generate realistic toxicological outcomes of ARVs based on actual environmental systems.

## 2. Materials and Methods

### 2.1. Chemicals

Efavirenz ((4S)-6-Chloro-4-(2-cyclopropylethynyl)-1,4-dihydro-4-(trifluoromethyl)-2H-3,1-benzoxazin-2-one; MW 315.67 g/mol; CAS Number: 154598-52-4), Tenofovir ((1R)-2-(6-Amino-9H-purin-9-yl)-1-methylethoxy]methyl] phosphonic acid monohydrate; MW 305.23 g/mol; CAS Number: 206184-49-8), dimethyl sulfoxide (DMSO; (CH_3_)_2_SO; MW 78.13 g/mol; CAS Number: 67-68-5; solubility: ≥22 mg/mL, purity: ≥99.7%), glacial acetic acid (CH_3_CO_2_H; MW: 60.05 g/mol; CAS Number: 64-19-7), perchloric acid (HClO_4_, MW: 100.46g/mol; CAS Number: 7601-90-3) and sulphuric acid (H_2_SO_4_; MW: 98.08 g/mol; CAS Number: 7664-93-9) were purchased from Sigma-Aldrich, South Africa. The physico-chemical properties of EFV and TFV are presented in Table S3.

### 2.2. Water Collection and Physicochemical Properties Determination

Water samples were collected from the Elands River (ER) (25°32′58.4″ S 28°33′53.4″ E) in Gauteng Province, South Africa. Water samples were collected only once in October 2019 to avoid variability in the physicochemical parameters of water. The collected water samples were filtered using 0.45 μm followed by a 0.2 μm pore size standard filter (Millipore) and were stored at 4 °C until use. The physicochemical characterization results of ER water are listed in Table S4. The presence of other chemical pollutants including pharmaceuticals in the natural water samples used were not quantified. The natural river water sampling site chosen was due to the unlikely presence of other pharmaceuticals or chemical pollutants based on its geographical location relative to the river source catchment zone. This is because the river water samples were taken on the catchment area site defined by no human settlements on the river upper side, thus limiting any likely presence of anthropogenic-based polluting chemicals (if any) including pharmaceuticals. It is in this context that the choice of the river water sampling site was considered to be less polluted due to no or very limited anthropogenic-based chemicals (if any) including pharmaceuticals.

### 2.3. Acute Toxicity Tests

Toxicity tests were performed following the DaphTox F magna™ kit procedure (MicroBioTests, 2006; Kit number DM361; Batch number DM130619). Thus, the methods followed were in agreement with standard protocols [49,50] recommended for acute toxicity tests. In brief, ephippia were rinsed with tap water, transferred into the hatching petri dish in 50 mL pre-aerated standard freshwater solution, and finally incubated at 20 ± 2 °C under a continuous light intensity of 6000 lux for 72 h. Thereafter, daphnia neonates (<24 h old) were fed spirulina 2 h before exposure tests were carried out. Four replicates of five neonates per nominal test concentration (62.5, 125, 250, 500, and 1000 µg/L), and control groups (ER water without ARVs) in 24 well-plates containing 10 mL of exposure media were incubated at 20 ± 2 °C in darkness for 48 h. Exposure concentrations were selected based on the previous studies. For example, stavudine (d4T) was investigated at 4.35 mg/L and did not induce immobilization of *D. magna* after 48 h [28]. Following exposure of two crustaceans, *D. magna* and *Artemia salina,* to abacavir (ABC) and acyclovir (ACV) ARVs, the 48 h-EC50 were ABC = >100 mg/L; ACV = 64.12 mg/L for *D. magna* and ABC = >100 mg/L; ACV = >100 mg/L for *A. salina*. In other previous work, immobilization of *A. salina* was observed after 24 h at very high concentrations of 30–180 mg/L tenofovir disoproxil fumarate (TDF) [12]. Thus, in this study the selected exposure concentrations were because, at the biochemical level, effects are likely to be induced at lower concentrations when compared to those that may cause immobilization as summarized in the above cited studies reported to date.

The water physicochemical parameters were established to be within the prescribed guidelines for the Daphnia toxicity tests [51], and results are summarized in Tables S4 and S5. The organisms were observed for immobilization (neonates’ inability to swim after 15 s of test plate agitation even if they still move their antenna) and mortality (absence of movement of the appendages and antennas) after 24 h and 48 h. The number of dead and/or immobile neonates was recorded. The test was considered valid if mortality and immobilization of the controls were ≤10 %.

### 2.4. Biochemical Assays

After 48 h, live *D. magna* were removed from the test plates. Organisms from the same test concentration or control groups were pooled together to yield enough biomass needed for the analysis of biochemical endpoints. The organisms were transferred to 1.5 mL microcentrifuge tubes and immediately analyzed for biomarkers related to detoxification (GST), and oxidative stress (CAT, LPO).

Fifteen exposed and non-exposed *D. magna* were homogenized in 300 µL of phosphate-buffered saline (PBS, pH 7.0) for 30 s using bead-based PowerLyzer^TM^ 24 homogenizer (MO BIO Laboratories, Inc.). The homogenate was centrifuged at 15,000× *g* for 15 min at 4 °C. Enzymatic activities of GST and CAT were measured in the freshly prepared supernatant. The CAT and GST activities were measured using Catalase Assay Kit (CAT100) and Glutathione S-Transferase Assay Kit (CS0410, Sigma-Aldrich Co., St Louis, MO, USA) commercial kits, respectively, following the specific manufacturer’s instructions. The absorbance was measured at wavelengths (λ) of 520 and 340 nm, respectively, for CAT and GST using a UV-Vis spectrophotometer (xMark Microplate Spectrophotometer, Bio-Rad, Hercules, CA, USA).

### 2.5. Lipid Peroxidation

Lipid peroxidation was determined by quantifying malondialdehyde (MDA) where lipid peroxides were measured using the generation of thiobarbituric acid (TBA). LPO was estimated using a Lipid Peroxidation (MDA) Assay Kit (MAK085, Sigma-Aldrich Co., St Louis, MO, USA) following the manufacturer’s protocol. Briefly, after 48 h of exposure, a pool of fifteen organisms from each test concentration or control group was homogenized in 300 µL of MDA Lysis Buffer containing 3 µL of butylated hydroxytoluene (BHT, 100×), and then centrifuged at 13,000× *g* for 10 min. Then, 600 µL of the TBA solution was added into each vial containing 200 µL standard or sample to form the MDA-TBA adduct. Both the prepared samples and the standard were incubated at 95 °C for 60 min and then cooled to room temperature in ice for 10 min. Absorbance was determined at λ = 532 nm using a UV-Vis spectrophotometer (xMark Microplate Spectrophotometer, Bio-Rad, Hercules, CA, USA).

### 2.6. Data Analysis

All measurements for biochemical markers were performed in three sub-replicates, and the results given as mean ± standard deviation (SD). Statistical analysis was conducted using GraphPad Prism Software version 9.1.1. (GraphPad Software, San Diego, CA, USA). The Kruskall–Wallis (nonparametric) test using R software was conducted on the averages of immobilization measures. One-way analysis of variance (ANOVA) followed by Dunnett’s post hoc test was used to evaluate the statistical differences of biochemical markers between ARVs-exposed samples and the controls. Differences between samples were considered statistically significant when *p* < 0.05.

## 3. Results and Discussion

### 3.1. Acute Toxicity Tests

**Figure 1:**
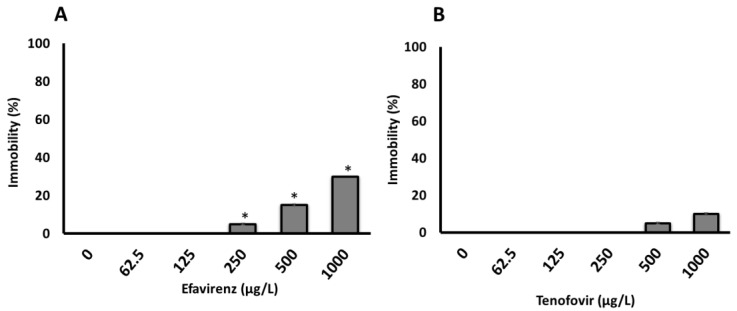
Immobilization of *D. magna* exposed to (**A**) EFV (*p*-value = 0.004025) and (**B**) TFV (*p*-value = 0.3313) for 48 h in ER water. Asterisks (*) represent significant difference between treatments (concentrations) from Kruskal-Wallis Test (* *p* <0.05). All data are the average of 4 replicates ± standard deviation.

The 100% survival of neonates and the lack of immobilization over 48 h in the control samples indicated that water without ARVs had no effect on daphnids’ health. The effects of the tested ARVs concentrations on *D. magna* immobilization were not observed after 24 h. However, a concentration-dependent immobilization was observed for both ARVs only after 48 h (Figure 1), with significantly more immobilization observed for EFV compared to TFV. The 48 h-E/LC_50_ was not calculated for the acute bioassays because the immobilization and/or mortality did not reach 50% even at the maximum concentration of 1000 µg/L for both ARVs tested in this study (Figure 1A, B and Table S6). This is consistent with previous findings where 48 h-EC_50_ values of abacavir (ABC) and acyclovir (ACV), both ARVs belonging to the NRTIs class, on *D. magna* were reported to be >100 and 64.12 mg/L, respectively [29]. The EC_10_ and EC_20_ were 467 and 537 µg/L (determined using Dr Fit Software [52]), respectively, for EFV, but were indeterminate for TFV.

At present, the highest measured environmental concentrations (MECs) of EFV and TFV detected in surface waters are 0.354 μg/L [3], and 0.25 μg/L [53], respectively. Following the WHO “treatment for all” recommendation [8], these concentrations and daily consumptions are expected to dramatically increase. Further, evidence indicates that TFV is widely used and rapidly excreted largely unchanged in the urine [54]. Thus, expected higher ARVs concentrations are likely to induce physiological level effects on daphnia. Although the tested ARVs showed no lethality, the observed immobilization is indicative of ARVs’ likelihood to induce long-term deleterious effects including at a sub-lethal level. The reason is that ARVs are considered to be “pseudo persistent” [54,55] as they are continuously released into the environment as evidenced by occurrence and fate data [2,4]. ARVs, in turn, may be transferred across trophic levels in the food chain, thereby altering the function of natural ecosystems.

Similar to current findings, previous studies on the toxicity of ARVs at apical endpoints indicated minimal or no deleterious effects on various aquatic organisms. For example, TFV (30–180 mg/L) in 3% saline solution (pH 8.0–9.0) induced immobilization in a microcrustacean *Artemia salina* after exposure for 24 h, with an inhibitory concentration (IC_50_) of 61.83 mg/L [12]. Further, the IC_50_ value of TFV for *A. salina* (61.83 mg/L) was lower than the estimated effective concentration (EC_50_) for a primary producer *Microcysti novacekii* (89 mg/L). The high sensitivity of *A. salina* compared to single-celled species, although the latter is known to be more sensitive to xenobiotics, may be due to the former’s ability to bioaccumulate xenobiotics [56]. Therefore, the disruption of aquatic invertebrates can induce potential adverse implications such as ecosystem imbalances. A concentration-dependent effect of EFV was reported in fish [5]. After exposure of *Oreochromis mossambicus* to 10.3 and 20.6 ng/L EFV for 96 h, results showed that the latter concentration induced liver damage, and an overall decline in fish health, but no effect was apparent for the former [5].

Effects for other ARVs, e.g., abacavir (ABC), acyclovir (ACV), stavudine (d4T), and zidovudine (ZDV), belonging to the NRTIs class, have been tested on *D. magna* [10,28,29]. Findings indicated that exposure of *D. magna* to ACV at 92.1 mg/L in OECD 211 medium for 21 d had no effects on mortality, reproduction, or population growth rate relative to the controls [10]. Similarly, exposure of *D. magna* to ZDV (4.5 mg/L) and d4T (4.35 mg/L) in ISO 6341 medium for 48 h induced no observable immobilization [28]. Exposure studies of *D. magna* and *A. salina* to ABC sulphate, and ACV over 48 h showed that *A. salina* (EC_50_: ABC ≥ 100 mg/L; ACV ≥ 100 mg/L) was less sensitive compared to *D. magna* (EC_50_: ABC ≥ 100 mg/L; ACV = 64.12 mg/L) [29].

Taken together, these findings point to limited data on the immobilization, mortality, and behavioral changes apical endpoints on daphnia exposed to ARVs to aid drawing firm conclusions. Therefore, comprehensive research studies on these endpoints using variant ARVs representing different classes are essential to offering insights into their likely acute and chronic toxicity. Additionally, based on the available data including those generated from the current study, findings indicated that most ARVs may induce no or a minimal effect on aquatic invertebrates at apical endpoints [10,29]. Hence, this raises the need to explore different endpoints at the biochemical level using enzymatic (CAT, GST) and non-enzymatic markers (LPO) known to be more sensitive even at relatively low exposure concentrations.

### 3.2. Effects on Biochemical Markers

Biochemical markers have been proposed as an early warning indicator of population-level effect(s) from sub-lethal exposure concentrations [57,58]. Most studies on the effects of ARVs on aquatic organisms have reported mostly apical endpoints, e.g., immobilization, growth inhibition, or survival and behavior [4,5,12,13,15,28,29]. From the published literature, only a single study reported the effects of ARVs on aquatic organisms using enzymatic biomarkers [14]. Fernández et al. [14] evaluated the toxicological effects of 3TC, d4T, and ZDV representing the NRTI class as well as NVP (NNRTI class) on *Rhinella arenarum* tadpoles using acetylcholinesterase (AChE) and GST enzymatic biomarkers. The authors documented evidence of short-term ARVs bioaccumulation in tadpoles with NVP exhibiting the highest bioaccumulation. Changes in GST activities were observed whereas AChE activity was similar relative to controls [14]. Findings indicated GST biomarkers to be more sensitive than AChE.

In this context, to date, there is a dearth of knowledge on the ecotoxicological effects of ARVs on non-target freshwater invertebrates at the biochemical level. Here, the present study seeks to generate information that can offer insights into the toxicological outcomes of ARVs and early responses of enzymatic biomarkers and further to contribute to the limited knowledge on the effects of ARVs on aquatic organisms at the biochemical level, especially using organisms at the lower trophic level in the food chain (e.g., *D. magna*). Thus, the present study investigated the effects of EFV and TFV on the biochemical biomarkers (CAT, GST, and LPO) of *D. magna*.

#### 3.2.1. Catalase Activity

**Figure 2:**
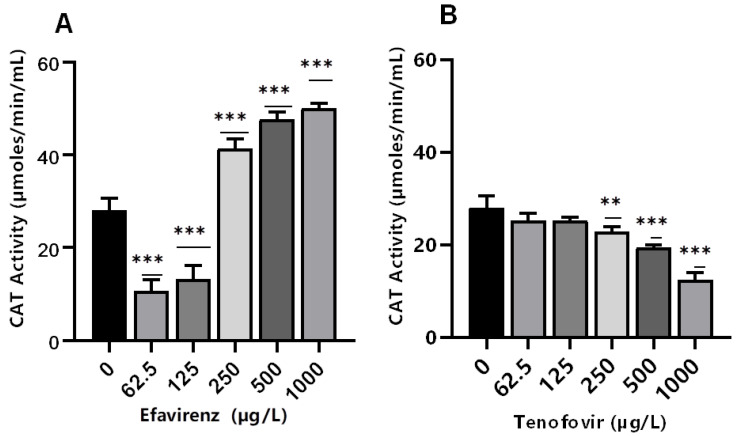
Catalase activity levels in *D. magna* exposed to (**A**) EFV, and (**B**) TFV for 48 h. Values are presented as mean ± standard deviation (*n* = 3); (Asterisks (*) denote significant differences (one-way ANOVA followed by Dunnett’s multiple comparisons test (** *p* ≤ 0.01, *** *p* ≤ 0.001)) between ARVs-exposed samples and the controls.

Antioxidant enzymes such as CAT are produced as a response to ROS production to balance the oxidative stress [39]. Catalase activity is produced in response to excessive H_2_O_2_ [44,45]. Here, CAT activity was significantly (*p* ≤ 0.001) altered at all concentrations for EFV compared to the controls (Figure 2A). Conversely, minimal effects on CAT activity were observed for TFV compared to controls (Figure 2B). The findings indicate that the exposure of daphnids to EFV at 250–1000 µg/L increased the production of ROS, which, in turn, induced the CAT activity, attributed to the removal of excess H_2_O_2_ [59,60,61]. The increase in this biomarker activity is indicative of the *D. magna* defense mechanism as a means to offset high oxidative stress linked to increased H_2_O_2_ production [43,58].

Conversely, CAT activity decreased with increasing concentration of TFV (*p* > 0.05 at 62.5–125 µg/L). However, a significant concentration-dependent decrease was observed at the higher exposure concentrations of 250 µg/L (*p* ≤ 0.01), and 500–1000 µg/L (*p* ≤ 0.001) (Figure 2B). These findings demonstrate that increasing TFV concentrations can result in intracellular ROS saturation, leading to oxidative stress and antioxidant system suppression [62]. The decrease in CAT activity can also be caused by excessive H_2_O_2_ production [63]. A possible explanation for the observed reduction in CAT can be attributed to high concentrations of exposure that exceeded the CAT’s tolerance range. Additionally, a reduction in CAT activity changes the redox status of the cells. This may result in excess generation of ROS or inadequate oxygen radical scavenging activity; free radical chain reactions are stimulated and interactions with protein, lipids, and nucleic acids could cause cellular damage [64]. CAT activity was not significantly affected by TFV. Results indicate that the levels of H_2_O_2_ resulting from putative oxidative stress were not paramount and hence did not require the overexpression of this specific biomarker [36].

#### 3.2.2. Glutathione S-Transferase Activity

**Figure 3:**
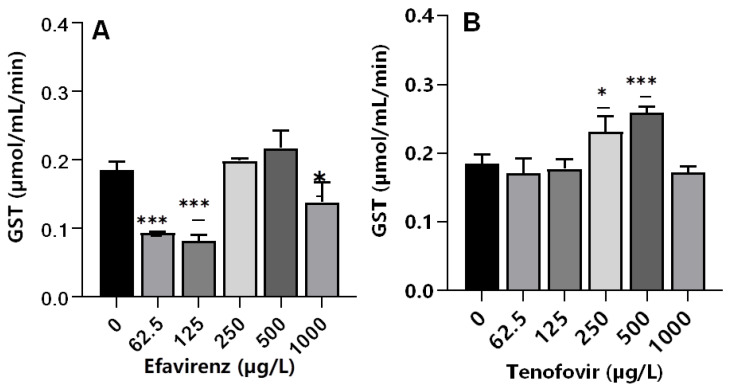
GST levels in *D. magna* exposed to (**A**) EFV, and (**B**) TFV for 48 h. Values are presented as mean ± standard deviation (*n* = 3); (Asterisks (*) denote significant differences (one-way ANOVA followed by Dunnett’s multiple comparisons test (* *p* <0.05, *** *p* ≤ 0.001)) between ARVs-exposed samples and the controls.

Glutathione *S*-transferases are multifunctional phase II versatile detoxification and xenobiotic metabolizing enzymes [65,66]. The conjugation of electrophilic compounds by GST with glutathione detoxifies harmful drugs and environmental chemicals; hence, GSTs are toxicologically important enzymes [67]. A significant decrease (*p* ≤ 0.001) in GST activity was observed at the lowest concentrations of 62.5 and 125 µg/L EFV, whereas a non-significant increase was apparent at 250 and 500 µg/L EFV (*p* > 0.05). At 1000 µg/L, a significant decrease in GST activity (*p* < 0.05) was observed compared to the control (Figure 3A). The decrease in GST activity was attributed to the direct inhibitory effect of EFV on enzymes or as a result of the generally impaired physiological state of an organism at sub-lethal exposure concentrations. GST activity increased significantly compared to the control only at 250 (*p* ≤ 0.05) and 500 µg/L (*p* ≤ 0.001) TFV, but remained unchanged relative to the control at all other exposure concentrations (Figure 3B). Oliveira et al. [36] reported insignificant changes in GST activity following exposure of *D. magna* to chlorpromazine and paracetamol at 0.01–1 mg/L, and a dose-dependent increase following exposure to propranolol. Hence, pharmaceuticals even of the same class could exert variant GST activity on crustaceans, e.g., *D. magna*.

The observed similar GST activity between exposed and non-exposed samples in this study demonstrated that, although CAT activity was observed to be affected, the imposed chemical stress was not strong enough to initiate other pathways. Similarly, following exposure of tadpoles to 3TC at 0.5, 1, 2, and 4 mg/mL, a significant increase in GST activity was only observed at the highest exposure concentration [14]. Notably, GST activity was only elevated at 250 and 500 µg/L for TFV but decreased at 62.5 and 125 µg/L EFV. This is in agreement with the findings of Martínez-Guitarte [68] where GST genes were either up-or down-regulated dependent on various factors including the chemical structure of the xenobiotic as well as exposure concentration and duration [68].

For both ARVs, GST activity at 1000 µg/L decreased compared to the control. This could be explained as a signal of overwhelmed antioxidant capacity caused by the higher concentrations of ARVs [60]. Following the exposure of *D. magna* to a fungicide boscalid (at 1.25, 2.5, 5, and 10 mg/L) for 48 h, authors observed dose-dependent changes in biochemical markers (CAT, GST, and LPO). Exposure to the two highest concentrations of 5 and 10 mg/L boscalid significantly decreased gene expression of SOD, GST, CYP4, and NRF1, but increased CAT gene expression [60]. The observed increase in GST activity is evidence of conjugation reaction with GSH for the elimination of TFV, and overproduction of ROS, which may lead to oxidative damage in *D. magna* following exposure to ARVs.

#### 3.2.3. Lipid Peroxidation

**Figure 4:**
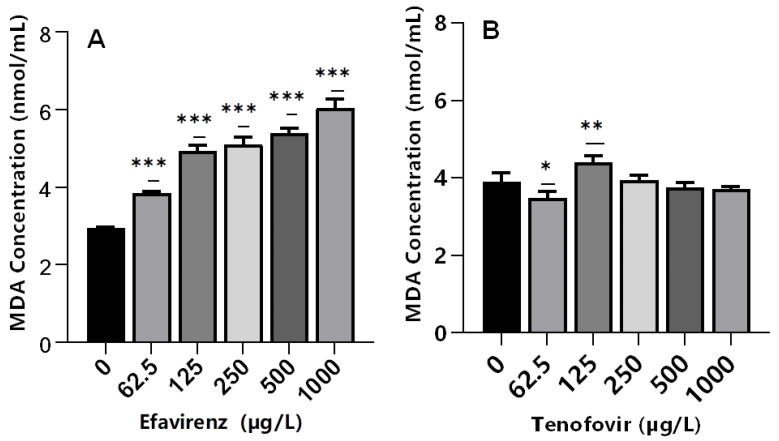
MDA levels in *D. magna* exposed to (**A**) EFV, and (**B**) TFV for 48 h. Values are presented as mean ± standard deviation (*n* = 3); (Asterisks (*) denote significant differences (one-way ANOVA followed by Dunnett’s multiple comparisons test (* *p* < 0.05, ** *p* ≤ 0.01, *** *p* ≤ 0.001)) between ARVs-exposed samples and the controls.

Lipid peroxidation is an indicator of excessive ROS generation associated with the production of highly reactive byproduct malondialdehyde (MDA). The MDA is considered an indicator of cell membrane damage due to free radicals causing severe oxidative stress [60,69]. This leads to impaired cellular functions and alterations in the physicochemical properties of cell membranes, which in turn could disrupt vital functions [70]. The exposure of daphnids to EFV significantly increased MDA levels (*p* ≤ 0.001) at all tested concentrations compared to the controls (Figure 4A); hence, evidence of LPO. The findings indicate that ROS may be associated with the metabolism of EFV and that antioxidant enzymes were insufficient to remove ROS, thereby inducing the peroxidation of membrane lipids. Further, these findings demonstrated that excessive ROS formation under short-term EFV stress may generate reactive products of LPO, but none were observed in the case of TFV.

Conversely, despite significant changes in CAT (250–1000 µg/L) and GST (250 and 500 µg/L) activities, MDA levels were not significantly different relative to the controls in TFV-treated samples at 250–1000 µg/L (Figure 4B). Recently, a similar effect was observed in *D. magna* exposed to silver and titanium dioxide nanoparticles [35]. This was attributed to the low concentrations of both nanoparticles types; hence, they were incapable of generating adequate ROS to trigger oxidative lipid damage [71]. Similarly, low concentrations of TFV used in this study may have not generated adequate ROS to trigger oxidative lipid damage.

Alternatively, the fact that LPO was unaffected at these concentrations, the induction of GST activity was most likely due to TFV detoxification, and not the detoxification of LPO by-products [33]. In addition, similar MDA levels for TFV- and non-exposed samples may indicate the efficacy of the antioxidant system to maintain intracellular ROS saturation. A significant (*p* < 0.05) decrease in MDA levels was observed in daphnids exposed to 62.5 µg/L (Figure 4B). Similar findings have been reported following exposure of mussels to ibuprofen [72], and fish to ivermectin [73], where elevated cellular antioxidant enzymes activities aided with depopulating ROS and reducing oxidative stress.

Overall, although variations in oxidative stress biomarkers have been reported to be influenced by abiotic factors (e.g., temperature, salinity, and dissolved oxygen content) [74,75,76], herein, water samples analysis before and during exposure (Tables S4 and S5) showed that all physicochemical parameters were within the guidelines for the Daphnia toxicity tests [51]. Thus, changes observed were attributed to the effect of ARVs. The sensitivity in enzymatic responses varied due to the function of each enzyme. For instance, at 62.5 and 125 µg/L EFV, both CAT and GST activities significantly decreased, thus being indicative of severe oxidative stress at these exposure concentrations [77]. However, MDA levels increased significantly at the same concentrations. Further, an increase in CAT activity and MDA levels in the 250–1000 µg/L EFV range can help to explain the increase in immobilization at these concentrations.

The increasing and decreasing patterns of antioxidant enzyme activity variations were found to be ARV type dependent. For instance, following the exposure of daphnids to 250–1000 µg/L EFV, CAT activity increased significantly. However, a decrease in CAT activity was observed in TFV-exposed samples at the same concentrations range. These findings demonstrated that the significant effects on antioxidant enzyme activities were more pronounced on EFV-exposed daphnids compared to TFV. This was attributed to the marked differences in ARVs physicochemical characteristics (e.g., water solubility, pKa, log Kow), structures, and mechanism of action. For example, pKa is a useful indicator parameter in physiological systems where dissociation affects the rate at which a chemical diffuses across the membranes resulting in uptake by organisms. This is because it influences lipophilicity, solubility, protein binding, and permeability of pollutants in focus, which in turn affects their ADME (Xie et al. 1991; Avdeef, 2001). Here, the pKa value of EFV is 10.2 (Hazardous Substances Data Bank; https://pubchem.ncbi.nlm.nih.gov/source/hsdb/7163: accessed on 15 January 2022), whereas for TFV it is 3.8 (DrugBank; https://www.drugbank.ca/drugs/DB14126: accessed on 15 January 2022). Hence, the lower pKa value for TFV points to likely deprotonation and higher solubility. As a result, TFV toxicity is expected to increase at lower pH values closer to the pKa. Thus, the low toxicity of TFV compared to EFV observed may be attributed to the higher pH of ER water used as an exposure medium in this study (8.12–8.28).

## 4. Environmental Implications

Here, multiple endpoints including mortality, immobilization and sub-lethal effect (biochemical biomarkers e.g., CAT, GST, LPO) were evaluated to understand the likely effects of ARVs on *D. magna*. CAT and GST enzymatic activities and MDA levels were elevated to detoxify H_2_O_2_ and protect Daphnia lipid and protein molecules against oxidative stress over 48 h. Results demonstrated that daphnia can actively use the antioxidant defense system to combat ARVs-induced oxidative stress. As a result, organisms are likely to allocate more energy to the oxidative stress defense system. This, in turn, may influence their growth and reproduction under chronic exposure conditions. For example, it was reported that, following exposure of *D. magna* to temperatures of 20 and 25 °C for 5–21 days, daphnids allocated more of their energy towards the oxidative stress defense system to improve self-maintenance. Consequently, both their growth and reproduction were compromised [78] and this has the potential to skew the ecological balance.

Following the “90-90-90” target and the “treatment for all” recommendations in ART initiation [8], this, in turn, will trigger a dramatic increase in ARVs emissions into the ecosystems globally. Similar to other pharmaceuticals (e.g., roxithromycin, propranolol), it is likely that ARVs present at a lower trophic level can be transferred to higher-level organisms as previously demonstrated by Ding and colleagues [79,80]. Thus, continuous exposure to ARVs as environmental pollutants may pose an existential ecological risk to *D. magna,* especially at sub-lethal levels.

Disruption of aquatic invertebrates, in turn, could induce potential adverse implications such as ecosystem imbalances. This is because *D. magna* are primary consumers and form a link between primary producers (e.g., algae) and tertiary consumers (e.g., fish) in the food chain. Understanding the effects of ARVs at lower trophic levels could help to predict their potential risks to human health. Our studies were carried out in river water as exposure media sought to be reflective of actual environmental systems as these are defined by complex and multifactorial influencing factors including pH, natural organic matter and variant electrolytes, among others. As the data on the ecotoxicity of ARVs are expected to increase, this can help with gaining a holistic overview of the implications of these chemical stressors at different levels of organizations across a broader ecological spectrum. As such, our data can support the development of tailor-made intervening mechanisms to promote a fine balance of protecting human health and ecological integrity as society races to address the HIV global pandemic.

## 5. Conclusions

Our findings revealed that *D. magna* is a sensitive bio-indicator to assess the ecotoxicity of ARVs in aquatic environments. The study offered insights into early (48 h) detection of the toxicological effects of ARVs on *D. magna* before more severe damage—e.g., mortality or population changes—can occur using biomarkers response endpoints. *D. magna* exhibited different responses depending on the ARV type as well as the biochemical marker tested. Findings demonstrated that both ARVs induced concentration-dependent immobility at the highest exposure concentrations of 250–1000 µg/L. At the biochemical level, toxicity was generally more pronounced in *D. magna* exposed to EFV compared to TFV as evidenced by significant changes in CAT and GST activities, and MDA levels relative to the controls. The ARVs had no effect on immobility at the lowest two exposure concentrations (62.5 and 125 µg/L), but findings at the biochemical level clearly demonstrated that oxidative stress responses were affected in *D. magna*.

Overall, our findings demonstrated that CAT, GST, and MDA may be involved in defense against oxidative stress induced by EFV and TFV exposure in daphnids. However, further studies investigating effects at the molecular level or on molecular markers (e.g., antioxidant enzyme-related genes), chronic and multigenerational effects are essential. This is to better understand the underlying toxicity mechanisms and the relationship between antioxidant enzyme system and oxidative damage of daphnids, and long-term effects in response to ARVs. This will provide insights into which genes are up-or down-regulated in response to ARVs exposure. Responses at the molecular level can act as early warning signals for the toxicological effects at the whole-organism level. Finally, we recommend reporting results based on ARVs concentrations measured during the study period as opposed to nominal concentrations.

## Data Availability

Not applicable.

## Supplementary Materials

The following supporting information can be downloaded at: https://www.mdpi.com/article/10.3390/toxics10080423/s1, Table S1: Classes and types of individual Food and Drug Administration-approved ARVs for clinical use; Table S2: Global measured environmental concentrations of ARVs in different matrices [3,22,53,81,82]; Table S3: Molecular Formula and physico-chemical properties of ARVs; Table S4: Physicochemical parameters of freshwater samples collected from Elands River; Table S5: Physicochemical properties of ER water during exposure recorded for the controls, lowest and highest exposure concentrations at 0 and 48 h; Table S6: Cumulative immobilization of *D. magna* exposed to variant concentrations of EFV and TFV.
