# Supplementary material for: Effects of Two Antiretroviral Drugs on the Crustacean Daphnia magna in River Water
Source: Toxics. 2022 Jul 28;10(8):423. doi: 10.3390/toxics10080423 (PMC9416331; doi:10.3390/toxics10080423)
Supplement: Supplementary file 1 [file toxics-10-00423-s001.zip › toxics-1782326-supplementary.pdf]

Article

Effects of Two Antiretroviral Drugs on the Crustacean *Daphnia magna* in River Water

Ntombikayise Mahaye and Ndeke Musee *

Emerging Contaminants Ecological and Risk Assessment (ECERA) Research Group, Department of Chemical Engineering, University of Pretoria, Pretoria, 0028, South Africa; mahaye.ntombi@gmail.com
* Correspondence: ndeke.musee@up.ac.za or museen2012@gmail.com

Table S1. Classes and types of individual Food and Drug Administration-approved ARVs for clinical use.

NRTIs	NNRTIs	Protease inhibitors	Fusion inhibitors	Entry inhibitors	Integrase inhibitors
Abacavir	Delavirdine	Atazanavir	Enfuvirtide	Enfuvirtide	Bictegravir
Didanosine	Doravirine	Darunavir		Maraviroc	Dolutegravir
Emtricitabine	Efavirenz	Fosamprenavir			Elvitegravir
Lamivudine	Etravirine	Indinavir			Raltegravir
Stavudine	Nevirapine	Lopinavir			
Tenofovir alafenamide	Rilpivirine	Ritonavir			
Tenofovir disoproxil fumarate		Saquinavir			
Zidovudine		Tipranavir			
		Cobicistat Nelfinavir			

Source: https://aidsinfo.nih.gov/understanding-hiv-aids/fact-sheets/21/58/fda-approved-hiv-medicines; NRTIs - nucleoside or nucleotide reverse transcriptase inhibitors; NNRTIs - non-nucleoside reverse transcriptase inhibitors

Table S2. Global measured environmental concentrations of ARVs in different matrices

Antiviral	Concentration (µg/L)	Location	Source	Reference
Efavirenz	34	South Africa	WWTP influent	Abafe et al., [1]
Efavirenz	20	South Africa	WWTP effluent	Abafe et al., [1]
Efavirenz	17.4	South Africa	WWTP influent	Schoeman et al., [2]
Efavirenz	7.1	South Africa	WWTP effluent	Schoeman et al., [2]
Efavirenz	1.37 ± 3.7	South Africa	WWTP influent	Mlunguza et al., [3]
Efavirenz	3.63 ± 2.0	South Africa	WWTP effluent	Mlunguza et al., [3]
Efavirenz	26.3 ± 0.08	South Africa	WWTP influent	Mlunguza et al., [3]
Efavirenz	37.3 ± 0.10	South Africa	WWTP effluent	Mlunguza et al., [3]
Efavirenz	1.67 ± 2.5	South Africa	WWTP influent	Mlunguza et al., [3]
Efavirenz	3.27 ± 1.6	South Africa	WWTP effluent	Mlunguza et al., [3]
Efavirenz	1.02 ± 3.1	South Africa	WWTP influent	Mlunguza et al.,[3]
Efavirenz	2.22 ± 0.8	South Africa	WWTP influent	Mlunguza et al., [3]
Efavirenz	3.64 ± 0.02	South Africa	WWTP effluent	Mlunguza et al., [3]
Efavirenz	17.4	South Africa	WWTP influent	Schoeman et al., [2]
Efavirenz	7.1	South Africa	WWTP effluent	Schoeman et al., [2]
Efavirenz	0.354	South Africa	Surface water	Rimayi et al., [4]
Tenofovir	0.11 ± 2.0	South Africa	Surface water	Mlunguza et al., [3]
Tenofovir	0.25 ± 2.4	South Africa	WWTP influent	Mlunguza et al.,[3]
Tenofovir	0.17 ± 2.6	South Africa	WWTP influent	Mlunguza et al., [3]
Tenofovir	0.10 ± 1.1	South Africa	WWTP influent	Mlunguza et al., [3]
Tenofovir	0.25	South Africa	Surface water	Wood et al., [5]
Tenofovir	0.16 – 0.19	South Africa	Surface water	Wood et al., [5]

Table S3. Molecular Formula and physico-chemical properties of ARVs.

Compound Name	Molecular Formula	Water solubility (mg/mL)	pKa	log Kow	Half-life (h)
Efavirenz	$C_{14}H_9ClF_3NO_2$	0.000093	10.2	4.7	40-55
Tenofovir	$C_9H_{14}N_5O_4P$	13.4	3.8	-	32

Source: https://pubchem.ncbi.nlm.nih.gov/

Table S4. Physicochemical parameters of freshwater samples collected from Elands River

Parameter	Unit	ER Water
pH	-	8,22
Electrical conductivity	mS/cm	231
Alkalinity	mg $CaCO_3$/L	101
Cl^-	mg/L	19,1
$SO4^{2-}$	mg/L	4,33
NO_3^-	mg/L	0,209
PO_4	mg/L	0,005
Ca^{2+}	mg/L	16,4
Mg^{2+}	mg/L	11,1

Na^{2+}	mg/L	18
K^{+}	mg/L	3,47
Al	mg/L	0,002
Fe^{3+}	mg/L	< 0,004
Cu^{2+}	mg/L	< 0,002
Zn^{2+}	mg/L	< 0,002
Hardness	mg $CaCO_3$/L	87
COD	mg/L	45,7
DOC	mg/L	6,5

The water chemistry analysis was done in a certified laboratory.

The pH, electrical conductivity (EC) (mS/cm), and oxygen concentration (mg/L) were measured from the control, as well as lowest and highest test concentrations for each ARV type at 0 and 48 h using HACH HQ40D portable multi-parameter meter (Loveland, Colorado, United States). Results are presented in Table S5.

Table S5. Physicochemical properties of ER water during exposure recorded for the controls, lowest and highest exposure concentrations at 0 and 48 h.

**Sample**	**Conc. (μg/L)**	**pH**		**EC (mS/cm)**		**O_2 Content (mg/L)**		**O_2 (%)**	
		0 h	48 h	0 h	48 h	0 h	48 h	0 h	48 h
EFV	0	7.86	7.58	257	290	7.96	7.78	103.3	103.5
	62.5	8.18	7.87	254	286	8.01	7.45	103.5	97.6
	1000	8.23	8.03	230	262	7.95	7.91	102.4	103.1
TFV	0	8.09	8.01	208	220	8.84	7.65	109.1	103.5
	62.5	8.24	8.16	220	238	8.81	7.76	115.1	105.9
	1000	8.28	8.12	196	218	8.57	7.75	113.1	105.0

Table S6. Cumulative immobilization of *D. magna* exposed to variant concentrations of EFV and TFV.

		Efavirenz				Tenofovir			
Concentration (μg/L)	Exposed organisms	24 h	48 h	Survival (%)	Immobility (%)	24 h	48 h	Survival (%)	Immobility (%)
0	20	0	0	100	0	0	0	100	0
62.5	20	0	0	100	0	0	0	100	0
125	20	0	0	100	0	0	0	100	0
250	20	0	1	95	5	0	0	100	0
500	20	0	3	85	15	0	1	95	5
1000	20	0	6	70	30	0	2	90	10

References

1. Abafe, O.A.; Späth, J.; Fick, J.; Jansson, S.; Buckley, C.; Stark, A.; Pietruschka, B.; Martincigh, B.S. LC-MS/MS Determination of Antiretroviral Drugs in Influents and Effluents from Wastewater Treatment Plants in KwaZulu-Natal, South Africa. *Chemosphere* **2018**, *200*, 660–670, doi:10.1016/j.chemosphere.2018.02.105.
2. Schoeman C; Mashiane M; Dlamini M; Okonkwo OJ Quantification of Selected Antiretroviral Drugs in a Wastewater Treatment Works in South Africa Using GC-TOFMS. *J Chromatogr Sep Tech* **2015**, *6*, 272, doi:10.4172/2157-7064.1000272.
3. Mlunguza, N.Y.; Ncube, S.; Mahlambi, P.N.; Chimuka, L.; Madikizela, L.M. Determination of Selected Antiretroviral Drugs in Wastewater, Surface Water and Aquatic Plants Using Hollow Fibre Liquid Phase Microextraction and Liquid Chromatography - Tandem Mass Spectrometry. *Journal of Hazardous Materials* **2020**, *382*, 121067, doi:10.1016/j.jhazmat.2019.121067.
4. Rimayi, C.; Odusanya, D.; Weiss, J.M.; de Boer, J.; Chimuka, L. Contaminants of Emerging Concern in the Hartbeespoort Dam Catchment and the UMngeni River Estuary 2016 Pollution Incident, South Africa. *Science of the Total Environment* **2018**, 10.
5. Wood, T.P.; Duvenage, C.S.J.; Rohwer, E. The Occurrence of Anti-Retroviral Compounds Used for HIV Treatment in South African Surface Water. *Environmental Pollution* **2015**, *199*, 235–243, doi:10.1016/j.envpol.2015.01.030.
